# GC/MS Analysis of Essential Oil and Enzyme Inhibitory Activities of *Syzygium cumini* (Pamposia) Grown in Egypt: Chemical Characterization and Molecular Docking Studies

**DOI:** 10.3390/molecules26226984

**Published:** 2021-11-19

**Authors:** Heba A. S. El-Nashar, Wagdy M. Eldehna, Sara T. Al-Rashood, Amal Alharbi, Razan O. Eskandrani, Shaza H. Aly

**Affiliations:** 1 Department of Pharmacognosy, Faculty of Pharmacy, Ain Shams University, Cairo 11566, Egypt; 2 Center of Drug Discovery Research and Development, Ain Shams University, Cairo 11566, Egypt; 3 Department of Pharmaceutical Chemistry, Faculty of Pharmacy, Kafrelsheikh University, Kafrelsheikh 33516, Egypt; 4 Department of Pharmaceutical Chemistry, College of Pharmacy, King Saud University, Riyadh 11451, Saudi Arabia; salrashood@ksu.edu.sa (S.T.A.-R.); amal.harbi@gmail.com (A.A.); Razan.a.esk@gmail.com (R.O.E.); 5 Department of Pharmacognosy, Faculty of Pharmacy, Badr University in Cairo, Cairo 11829, Egypt; shaza.husseiny@buc.edu.eg

**Keywords:** acetylcholinesterase, α-amylase, *α*-glucosidase, cytotoxicity, essential oil, molecular docking, *Syzygium cumini*, Pomposia

## Abstract

*Syzygium cumini* (Pomposia) is a well-known aromatic plant belonging to the family Myrtaceae, and has been reported for its various traditional and pharmacological potentials, such as its antioxidant, antimicrobial, anti-inflammatory, and antidiarrheal properties. The chemical composition of the leaf essential oil via gas chromatography–mass spectrometry (GC/MS) analysis revealed the identification of fifty-three compounds representing about 91.22% of the total oil. The identified oil was predominated by *α*-pinene (21.09%), followed by *β*-(E)-ocimene (11.80%), D-limonene (8.08%), *β*-pinene (7.33%), and *α*-terpineol (5.38%). The tested oil revealed a moderate cytotoxic effect against human liver cancer cells (HepG2) with an IC_50_ value of 38.15 ± 2.09 µg/mL. In addition, it effectively inhibited acetylcholinesterase with an IC_50_ value of 32.9 ± 2.1 µg/mL. Furthermore, it showed inhibitory properties against α-amylase and α-glucosidase with IC_50_ values of 57.80 ± 3.30 and 274.03 ± 12.37 µg/mL, respectively. The molecular docking studies revealed that (*E*)-*β*-caryophyllene, one of the major compounds, achieved the best docking scores of −6.75, −5.61, and −7.75 for acetylcholinesterase, α-amylase, and α-glucosidase, respectively. Thus, it is concluded that *S. cumini* oil should be considered as a food supplement for the elderly to enhance memory performance and for diabetic patients to control blood glucose.

## 1. Introduction

*Syzygium cumini* (L.) is an aromatic evergreen plant belonging to the family Myrtaceae, originating in Asia and widely distributed in America [1]. It is commonly known as jamum or jambul in Asia and as jambolão or jamelão in Brazil [2]. Traditionally, it has acquired great value in the Ayurveda and Unani systems of medication for possessing various treatment applications such as digestive, carminative, anthelmintic, antiulcer, bronchitis, antiasthma, antiallergic, diuretic, antiscorbutic, and wound healing [3,4]. Further, a wide range of pharmacological studies have been carried out on *S. cumini* that have proven its therapeutic potential as an antioxidant, antidiabetic, antimicrobial, anti-HIV, anti-inflammatory, and antidiarrheal [5,6,7]. Phytochemical investigations have shown that the plant is rich in flavonoids, tannins, anthocyanins, triterpenoids, essential oil, vitamins, and fatty acids [8,9]. Interestingly, the fruits of *Syzygium cumini* are purplish black in color with a pleasant odor, are edible with a high nutritional value, and are involved in many food products such as ice cream, jam, jellies, and yogurt [10]. Previously, the oil isolated from *S. cumini* leaves growing in Brazil exerted antioxidant, molluscicidal, and leishmanicidal effects [11,12].

Recently, the inhibition of key enzymes involved in the pathogenesis of disease has become a most effective therapeutic technique [13]. For instance, the most widely accepted strategy for the management of Alzheimer’s disease is to inhibit acetylcholinesterase (AChE) [14]. Furthermore, the inhibition of carbohydrate-metabolizing enzymes such as *α*-amylase and *α*-glucosidase is an effective mechanism to control hyperglycemia in diabetic patients [15]. Interestingly, synthetic drugs such as galantamine and acarbose are well-developed enzyme inhibitors in the pharmaceutical market for the management of Alzheimer’s disease and diabetes mellitus, but these drugs are known for side effects, such as hepatic injury and bowel disorders [16,17]. Consequently, medicinal plants have great value in an era of natural enzyme inhibitors to overcome the struggles of synthetic drugs [18]. Several essential oils from the genus *Syzygium* exploit enzyme inhibitory activities [19]. For example, *S. aromaticum* oil has shown remarkable antidiabetic activity via its inhibitory effect against *α*-amylase [20].

Molecular docking was chosen as the most suitable method to assess the underlying mechanism of inhibitory action for the pharmacologically active components, thus helping us understand the interactions of the enzyme with the major oil components [21]. In turn, molecular modeling provides information about the most appropriate geometry and binding affinity of the tested components (ligand) to the active site of the enzymes (target macromolecules) [22].

The present study was designed to investigate the chemical composition and enzyme inhibitory properties of the essential oil isolated from *Syzygium cumini* (L.) leaves grown in Egypt. The enzyme inhibitory assays were evaluated against AChE, *α*-amylase, and *α*-glucosidase. An added objective was to evaluate the binding affinities between the characterized major oil components and the tested enzymes using molecular docking studies.

As the essential oils are mostly safe with few complications, they can be a valuable non-medicinal option or coupled with conventional care for some health conditions, provided safety and quality issues are taken into consideration [23].

## 2. Results and Discussion

### 2.1. GC/MS Analysis of Essential Oil

**Figure 1:**
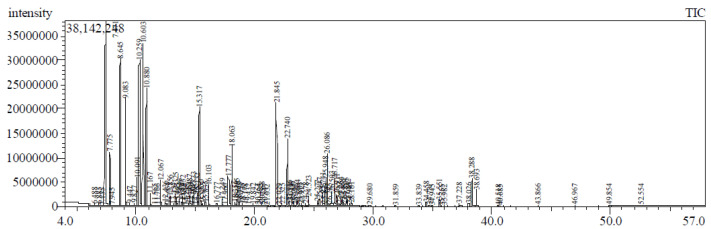
GC chromatogram of essential oil isolated from *Syzigium cumini* leaves grown in Egypt.

The GC/MS analysis revealed identification of fifty-three compounds, representing about 91.22% of total oil as shown in Figure 1 and Table 1. The major components of the oil were found to be α-pinene (21.09%), followed by *β*-(E)-ocimene (11.80%), D-limonene (8.08%), *β*-pinene (7.33%), α-terpineol (5.38%), (E)-*β*-caryophyllene (4.51%), and myrcene (3.90%). The oil was predominated by hydrocarbon monoterpenes representing about 61.82%, followed by oxygenated monoterpenes (15.17%), hydrocarbon sesquiterpenes (8.18%), and oxygenated sesquiterpenes (6.02%).

Previous reports concerning the essential oil of *Syzygium* revealed differences in its chemical composition relative to its geographical collection area. The major compounds of the essential oil of leaves of *S. cumini* (L.) Skeels, collected from the southwestern region of Brazil are sesquiterpenes, namely, *α*-caryophyllene and *β*-caryophyllen, *α*-terpineol, and iso-caryophyllene, along with caryophyllenyl alcohol and oxide [1]. The essential oil of *S. cumini* collected from India showed *τ*-cadinol and *τ*-muurolol to be the major compounds, followed by *τ*-globulol, caryophyllene, *δ*-cadinene, and *α*-pinene [24]. The collected S. cumini leaves from Pakistan revealed the major compounds to be *β*-farnesene, cis-*β*-ocimene, terpinen-4-ol, fenchol, *β*-myrcene, and *γ*-cadinene [25]. Another report revealed that the principal components of the essential oil isolated from *S. jambos* collected from Brazil to be (*E*)-caryophyllene, *α*-humulene, *α*-zingibirene, hydroxytoluene butylated, caryophyllene alcohol, caryolan-8-ol, caryophyllene oxide, thujopsan-2-*α*-ol, and *n*-heneicosane [26].

Previous studies of the essential oil content of *S. cumini* leaves from Egypt revealed components that were comparable to our results, *α*-pinene, *β*-pinene, trans-caryophyllene, and *α*-limonene [12]. The chemical constituents of another species collected from a private garden on the Cairo–Alexandria desert road, Egypt, namely, *S. aqueum* and *S. samarangense,* showed differences from *S. cumini*, whereby the essential oil of leaves of *S. aqueum* showed a high percentage of *α*-selinene followed by *β*-caryophyllene and *β*-selinene, and Germacrene D was found to be the major constituent in *S. samarangense* essential oil [27]. From these previous reports, it is obvious that the essential oil composition shows variability between different *Syzygium* species along with their geographical distribution.

### 2.2. Cytotoxic Activity

Essential oils are well known for their richness with oxygenated and non-oxygenated components, such as monoterpenes and sesquiterpenes, and previous reports have revealed their impact on several cancer cell lines [28,29]. The results of the cytotoxicity of the essential oil of *S. cumini* leaves revealed its inhibitory effect on human liver cancer cells (HepG2), with an IC_50_ value of 38.15 ± 2.09 µg/mL as compared to staurosporine (IC_50_ = 8.637 ± 0.47 µg/mL) as a reference drug.

In agreement with our results, *α*-pinene, one of the major components of *S. cumini* oil, showed inhibition toward hepatoma carcinoma BEL-7402 cells with an inhibitory rate of 79.3% in vitro and 69.1% in vivo through the suppression of growth of tumor cells in tumor-bearing mice. Additionally, *α*-pinene induced a significant increase in the G2/M population of the hepatoma cell line (BEL-7402) [30]. One of the major components of *S. cumini* oil is *β*-caryophyllene, which has shown potent cytotoxicity towards human oral squamous (OEC-M1) cells, human hepatocellular carcinoma (J5) cells, human lung adenocarcinoma (A549) cells, human colon (HT-29) cells, human melanoma (UACC-62) cells, and human leukemic (K562) cells with IC_50_ values of 24.0, 111.2, 31.3, 9.8, 3.2, and 4.6 μg/mL, respectively (Su and Ho 2013). In addition, it has exhibited cytotoxicity towards MCF-7, MDA-MB-468, UACC257, A549, Hela, and HT-29 cancer cell lines [31,32].

### 2.3. Acetylcholinesterase Inhibition

The acetylcholinesterase enzyme is responsible for the hydrolysis of acetylcholine, which is considered a key enzyme in the treatment of Alzheimer’s disease. Several plants have been reported for their inhibitory activity against acetylcholinesterase [33,34,35]. The essential oil of *S. cumini* showed moderate inhibitory ability against AChE with an IC_50_ value of 32.90 ± 2.10 µg/mL as compared to donepezil (IC_50_ = 7.89 ± 1.30 µg/mL) as a reference drug. 

The essential oil of *S. cumini* leaves showed promising anti-cholinesterase inhibitory activity that could be attributed to the presence of some volatile compounds such as monoterpenes: *α*-pinene, *β*-pinene, and *β*-(*E*)-ocimene being major compounds; along with the presence of sesquiterpenes, such as (*E*)-*β*-caryophyllene, which has been supported by previous investigations [36,37,38,39]. Mohamed et al. reported on the antioxidant activity of the essential oil of *S. cumini* leaves using ferric reducing power (FRAP) assays [12]. They showed that the highest ferric reducing power property was 0.47 mg/100 mg of essential oil, which correlated with the presence of a mixture of monoterpene hydrocarbons and oxygen containing mono- and sesquiterpenes. In addition, *S. cumini* leaves have been reported for their traditional use as a natural antioxidant agent [12]. The essential oil of *S. cumini* leaves isolated from the mature trees in Pakistan showed DPPH radical scavenging activity with an IC_50_ value of 1.2 mg/mL [24]. The potential activity of the essential oil components of *S. cumini* leaves, as new AChE inhibitors and antioxidants, could be considered a potential strategy for treating and decreasing the progress of Alzheimer’s disease [39,40].

### 2.4. α-Amylase and α-Glucosidase Inhibition

Inhibition of the *α*-amylase enzyme has been reported in previous studies to be one of the effective approaches for diabetes management through reduction in the postprandial hyperglycemia associated with type 2 diabetes mellitus [41,42]. Regarding our results, the essential oil of *S. cumini* leaves showed α-amylase inhibitory ability with an IC_50_ value of 57.80± 3.30 µg/mL as compared to acarbose (IC_50_ = 34.71 ± 2.30 µg/mL) as a reference drug. In addition, it showed moderate α-glucosidase inhibitory properties with an IC_50_ value of 274.03 ± 12.37 µg/mL as compared to acarbose (IC_50_ = 138.76 ± 7.59 µg/mL).

The antidiabetic effects of the *S. cumini* volatile components as enzymes inhibitors could be attributed to the presence of oxygenated compounds, such as monoterpene and sesquiterpene, which can bind non-selectively to amino and sulfhydryl groups of enzymes and cause a conformational change and loss of activity [43]. Previous reports concerning the seed extract of *S. cumini* in south India have shown an *α*-amylase enzyme inhibitory effect of up to 95.4% [44]. In accordance with these previous reports, we found that *α*- and *β*-pinene showed *α*-amylase inhibitory ability that suggests their responsibility for the observed inhibitory activity of the essential oil of *S. cumini* [45,46].

### 2.5. Molecular Docking

**Figure 2:**
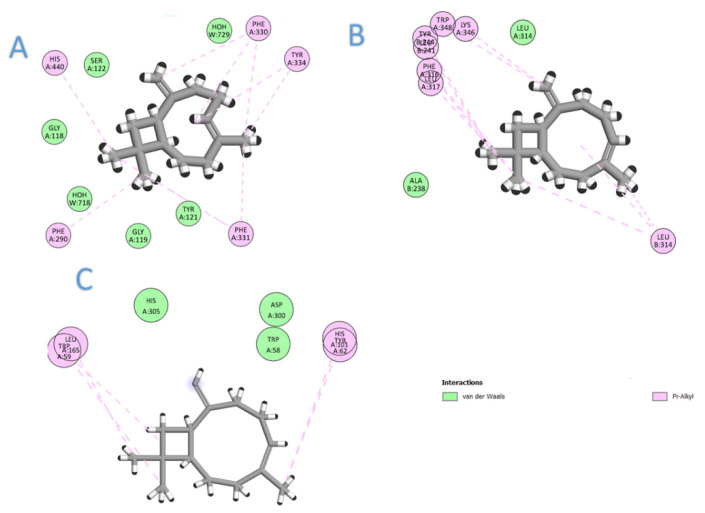
The 2D interaction diagram of (*E*)-*β*-caryophyllene with the three targets: (**A**) acetylcholinesterase; (**B**) *α*-glucosidase; (**C**) *α*-amylase.

**Figure 3:**
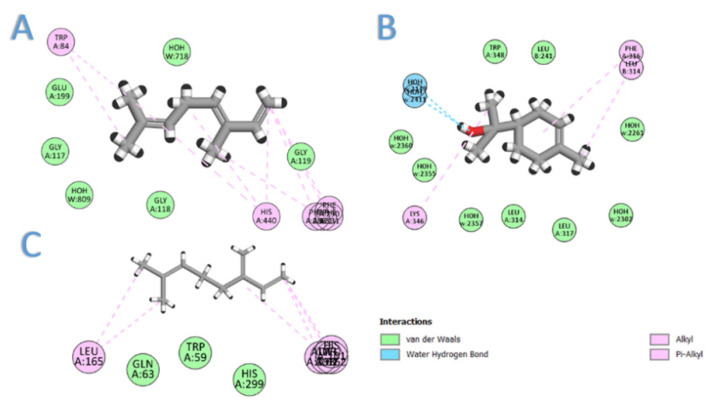
The 2D interaction diagram of: (**A**) *β*-(*E*)-ocimene with acetylcholinesterase; (**B**) *α*-terpineol with *α*-glucosidase; (**C**) myrcene with *α*-amylase.

In this section, we aimed to identify the molecular mechanism of action as well as the binding mode of the identified compounds. Therefore, the crystal structures of the acetylcholinesterase, *α*-amylase, and *α*-glucosidase were downloaded from the PDB and prepared for docking. The docking protocol was roughly validated by re-docking each o-crystalized ligand into its corresponding active site. The calculated RMSD between the co-crystalized pose and the docked pose was 0.53, 0.82, and 0.76 for AChE, *α*-amylase, and *α*-glucosidase, respectively, highlighting the validity of the docking. To benchmark our docking results so far, the docking score of each crystal reference was taken into consideration when comparing the docking scores of the seven selected compounds. As depicted in Table 2, all the major compounds in the isolated oil achieved favorable-accepted scores with the three targeted enzymes. Taking into account the hydrophobic nature of the isolated oil—composed entirely from hydro-carbonic compounds—all the interactions formed between any compounds within the three targets were found to be hydrophobic in nature (see Figure 2). Among the tested seven compounds, (*E*)-*β*-caryophyllene was the best compound to interact with the three enzymes, achieving docking scores of −6.75, −5.61, and −7.75 for AChE, *α*-amylase, and *α*-glucosidase, respectively. On the second rank after (*E*)-*β*-caryophyllene came *β*-(*E*)-ocimene, myrcene, and *α*-terpineol for AChE, *α*-amylase, and *α*-glucosidase, respectively (see Figure 3).

In conclusion, from the major oils, (*E*)-*β*-caryophyllene was the best compound to interact with the three enzymes, achieving docking scores of −6.75, −5.61, and −7.75 for AChE, *α*-amylase, and *α*-glucosidase, respectively. Compounds achieved acceptable scores with these three enzymes through the establishment of many hydrophobic interactions. It is worth mentioning that no compound was able to single-handedly overcome the scores achieved by any co-crystalized ligand, which highlights that their biological effects come from synergistic contributions from all the compounds.

## 3. Materials and Methods

### 3.1. Plant Material

Fresh leaves of *Syzygium cumini* (L.) were collected from a private garden in the Abo-Zabal area (N 30°17′43.386′′, E 31°22′27.9804′′), Qualiobya, Egypt, in February 2021. They were kindly authenticated by taxonomy specialist engineer, Therease Labib, the taxonomy specialist at El-Orman Botanical Garden, Giza, Egypt. A voucher specimen, PHG-P-SC-348, was deposited at the Pharmacognosy Department, Faculty of Pharmacy, Ain Shams University.

### 3.2. Isolation of the Essential Oil

The fresh leaves were finely cut and hydrodistilled for 5 h using a Clevenger apparatus. After hydrodistillation, the essential oil was isolated and kept in a sealed glass tube at −4 °C until GC/MS analysis.

### 3.3. Gas Chromatography–Mass Spectrometry (GC/MS)

The GC/MS analysis of the resulting oil was carried out at the Department of Medicinal and Aromatic Plants Research, National Research Centre, with the following specifications. Instrument: a TRACE GC Ultra Gas Chromatographs (THERMO Scientific Corp., Waltham, MA, USA), coupled with a thermo mass spectrometer detector (ISQ Single Quadrupole Mass Spectrometer). The GC–MS system was equipped with a TG-5MS column (30 m × 0.25 mm i.d., 0.25 μm film thickness). Analysis was carried out using helium as carrier gas at a flow rate of 1.0 mL/min and a split ratio of 1:10 using the following temperature program: 80 °C for 2 min; rising 5.0 °C/min to 300 °C; and held for 5 min. The injector and detector were held at 280 °C, and 0.2 μL of diluted samples (1:10 hexane, *v*/*v*) was injected. Mass spectra were obtained by electron ionization (EI) at 70 eV, using a spectral range of *m*/*z* 35–500.

### 3.4. Identification of Oil Components

The components of the essential oil were characterized by comparison of their GC/MS spectra, fragmentation patterns, and retention indices with those reported in the literature data [28,47]. The retention indices were calculated relative to a homologous series of *n*-alkanes (C8-C28) injected under the same conditions.

### 3.5. Assessment of Cytotoxic Activity

The cytotoxicity of the essential oil of *S. cumini* leaves was evaluated against human liver cancer cells (HepG2) using MTT (3-(4,5-dimethylthiazolyl-2)-2,5-diphenyltetrazolium bromide) assay [28,48,49]. The cell lines were obtained from American Type Culture Collection. The cell viability was evaluated based on the reduction in MTT by the metabolically active cells using staurosporine as a reference drug (Sigma-Aldrich chemicals). The results were expressed as the concentration that induces 50% of maximum inhibition of cell proliferation (IC_50_) from graphic plots of the dose–response curve for each concentration using Graphpad Prism software (San Diego, CA, USA).

### 3.6. Assessment of Enzyme Inhibitory Activities

#### 3.6.1. Acetylcholinesterase Inhibition Assay

Cholinesterase (ChE) inhibitory activity was evaluated using acetylcholinesterase activity colorimetric assay kit (Bio-vision company; K197-100), following Ellman’s method as previously described [17]. About 10 μL of essential oil was mixed with colorimetric substrate (DTNB) and AChE solution in Tris–HCl buffer (pH 8.0) in a 96-well microplate and incubated for 10–15 min at room temperature away from light. The reaction was based on the ability of an active human AchE enzyme to hydrolyze the provided colorimetric substrate, producing a yellow compound. Similarly, a blank was prepared by addition of a sample solution to all reaction reagents without enzyme solution. Donepezil was used as the positive control (Sigma-Aldrich, St. Louis, MO, USA). The absorbance of the sample, blank, and standard was measured at 412 nm after 10 min incubation at room temperature. The absorbance of the blank was subtracted from the value of the sample, and the results were recorded as IC_50_.

#### 3.6.2. α-Amylase Inhibition Assay 

The inhibitory activity of the tested oil was performed according to the standard method with minor modifications [50]. The enzyme solution was prepared by dissolving α-amylase in 20 mM phosphate buffer (PH = 6.9) at a concentration of 0.50 mg/mL. Then, 1 mL of the tested oil of various concentrations (1000–7.81 μg/mL) and 1 mL of enzyme solution were mixed and incubated at 25 °C for 10 min. After incubation, 1 mL of starch (0.50%) solution was added to the mixture and further incubated at 25 °C for 10 min. The reaction was then stopped by adding 2 mL of dinitro salicylic acid (3,5-dinitrosalicylic acid, color reagent) and heating the reaction mixture in a boiling water bath for 5 min. After cooling, the absorbance was measured colorimetrically at 565 nm. Acarbose was used as a standard drug. The inhibition percentage was calculated using the given formula: % inhibition = (1 − As/Ac) × 100, where As was the absorbance of the tested compound, and Ac was the absorbance of the control reaction (containing all reagents except the test sample). The IC_50_ value was defined as the concentration of the *α*-amylase inhibitor to inhibit 50% of its activity under the assay’s conditions.

#### 3.6.3. α-Glucosidase Inhibition Assay

The *α*-glucosidase inhibitory activity was performed according to the previously reported method using BioVision’s α-glucosidase inhibitor screening kit (K938-100) [17]. About 10 µL of leaf oil was mixed with glutathione (10 µL), α-glucosidase solution (10 µL) in phosphate buffer (pH 6.8), and PNPG (4-nitrophenyl-α-D-glucopyranoside) (10 µL) in a 96-well microplate and incubated for 15–20 min at room temperature. Similarly, a blank was prepared by adding the sample solution to all reaction reagents without α-glucosidase solution. The reaction utilized the ability of an active α-glucosidase to cleave a synthetic substrate (PNPG), releasing a chromophore (p-nitrophenol; OD = 410 nm). The reaction was then stopped with the addition of sodium carbonate (50 µL, 0.2 M). The absorbance of the tested oil and blank was read at 410 nm. The absorbance of the blank was subtracted from the values of the tested oil, and the results were reported as IC_50_.

### 3.7. Molecular Docking Studies

The crystal structures of acetylcholinesterase, *α*-amylase, and *α*-glucosidase were downloaded from the protein data bank (www.pdb.org) with the following IDs 1DX6, 4GQR, and 2V3E [51,52,53], respectively. All the docking studies were conducted using MOE 2019 [54], and the results were visualized by the open-source Discovery studio. Firstly, all the target enzymes, co-crystalized ligands, and the isolated compounds were prepared using the default settings. The active site of each target was determined from the binding of the corresponding co-crystalized ligand. Prior to commencing the docking of the seven identified compounds, a pose retrieval step for the co-crystalized ligands was performed and followed by RMSD calculation. Finally, the identified compounds were docked at the predetermined active site of the three target enzymes.

## 4. Conclusions

In our study, we described the detailed chemical composition of leaf oil isolated from *S. cumini* grown in Egypt. The tested oil was found to be rich in *α*-pinene (21.09%), *β*-(*E*)-ocimene (11.80%), D-limonene (8.08%), *β*-pinene (7.33%), *α*-terpineol (5.38%), and (*E*)-*β*-caryophyllene (4.51%). Further, we investigated its cytotoxic effects against the human liver cell line. In addition, we explored its effective inhibitory properties against acetylcholinesterase, α-amylase, and α-glucosidase. Our findings show that we should consider this oil for use as a food supplement or adjuvant therapy for the elderly to enhance memory performance and for diabetic patients to control blood glucose. Furthermore, the essential oils exhibited stronger toxicity towards the different pathogens documented in the literature [55,56,57,58], making the essential oil and its constituents, *α*-pinene, *β*-caryophyllene, and *α*-terpineol, a good candidate as an antimicrobial, antifungal, and insecticidal agent. Further in vivo neuroprotective and antihyperglycemic investigations are recommended to construct a molecular mechanistic profile for the isolated essential oil in the management of Alzheimer’s disease and diabetic mellitus. Lastly, without further investigations regarding its toxicity, the in vitro results in the present study should not be considered as encouraging the use of *S. cumini* essential oil as a herbal medicinal product.

## Figures and Tables

**Table 1:** Chemical composition (%) of essential oil identified from *Syzigium cumini* leaves grown in Egypt using GC/MS analysis.

No.	Compound	Retention Time (Rt)	Molecular Formula	Retention Index		Peak Area (%)
				Exp.	Rep.	
1	Bornylene	6.488	C_10_H_16_	885	890	0.02
2	*α*-Thujene	7.135	C_10_H_16_	908	911	0.02
3	*α*-Pinene	7.470	C_10_H_16_	921	924	21.09
4	Camphene	7.775	C_10_H_16_	930	932	1.86
5	2,4(10)-Thujadiene	7.945	C_10_H_16_	938	943	0.02
6	*β*-Pinene	8.645	C_10_H_16_	963	963	7.33
7	Myrcene	9.085	C_10_H_16_	979	983	3.90
8	*α*-Phellandrene	9.445	C_10_H_16_	992	998	0.09
9	*α*-Terpinene	9.825	C_10_H_16_	1006	1012	0.11
10	o-Cymene	10.090	C_10_H_14_	1014	1018	1.24
11	D-Limonene	10.260	C_10_H_16_	1020	1021	8.08
12	*β*-(*E*)-Ocimene	10.605	C_10_H_16_	1031	1035	11.80
13	*α*-(*E*)-Ocimene	10.880	C_10_H_16_	1040	1042	4.82
14	*γ*-Terpinene	11.165	C_10_H_16_	1049	1052	0.48
15	Isoterpinolene	12.065	C_10_H_16_	1078	1082	0.96
16	Fenchol	12.855	C_10_H_18_O	1103	1110	0.47
17	(*E*)-Sabinene hydrate	13.135	C_10_H_18_O	1112	1104	0.12
18	Neo-allo-ocimene	13.325	C_10_H_16_	1118	1123	0.58
19	4(10)-Thujen-3-ol	13.645	C_10_H_16_O	1128	1137	0.32
20	Thujol	13.930	C_10_H_18_O	1135	1138	0.31
21	Pinocarvone	14.380	C_10_H_14_O	1152	1158	0.09
22	(−)-Borneol	14.485	C_10_H_18_O	1155	1163	0.43
23	Terpinen-4-ol	14.835	C_10_H_18_O	1167	1172	0.53
24	Crypton	15.130	C_9_H_14_O	1176	1177	0.60
25	*α*-Terpineol	15.315	C_10_H_18_O	1182	1184	5.38
26	(−)-Myrtenol	15.450	C_10_H_16_O	1187	1189	0.30
27	Carveol	15.625	C_10_H_16_O	1192	1200	0.15
28	(+)-Verbenone	15.835	C_10_H_14_O	1199	1201	0.34
29	Fenchyl acetate	16.105	C_12_H_20_O_2_	1208	1212	0.74
30	*p*-Cumic aldehyde	16.775	C_10_H_12_O	1232	1237	0.08
31	Phellandral	17.775	C_10_H_16_O	1267	1272	1.46
32	Bornyl acetate	18.065	C_12_H_20_O_2_	1277	1277	2.35
33	(−)-*E*-Pinocarvyl acetate	18.355	C_12_H_18_O_2_	1287	1293	0.18
34	(+)-*Z*-Verbenol, acetate	18.465	C_12_H_18_O_2_	1291	1294	0.29
35	Carvacrol	18.615	C_10_H_14_O	1296	1298	0.21
36	Myrtenyl acetate	19.180	C_12_H_18_O_2_	1316	1323	0.06
37	*α*-Terpinyl acetate	19.430	C_12_H_20_O_2_	1324	1328	0.02
38	*α*-Copaene	20.590	C_15_H_24_	1365	1370	0.16
39	(*E*)-*β*-Caryophyllene	21.845	C_15_H_24_	1410	1412	4.51
40	*α*-Humulene	22.740	C_15_H_24_	1445	1447	2.71
41	Alloaromadendrene	22.915	C_15_H_24_	1452	1455	0.19
42	Eudesma-4(14),11-diene	23.605	C_15_H_24_	1479	1479	0.30
43	*α*-Selinene	23.805	C_15_H_24_	1487	1490	0.15
44	*α*-Amorphene	23.915	C_15_H_24_	1491	1497	0.07
45	*α*-Farnesene	24.280	C_15_H_24_	1505	1508	0.09
46	Palustrol	25.740	C_15_H_26_O	1562	1563	0.46
47	(−)-Spathulenol	25.950	C_15_H_24_	1570	1572	1.22
48	Caryophyllene oxide	26.085	C_15_H_24_	1576	1577	2.32
49	Epiglobulol	26.500	C_15_H_26_O	1592	1589	0.76
50	Humulenol-II	27.255	C_15_H_24_	1624	1632	0.39
51	Longipinocarveol, trans-	27.360	C_15_H_24_	1628	1634	0.42
52	(*E*)-Guai-11-en-10-ol	27.705	C_15_H_26_O	1643	1654	0.45
53	Hibaene	33.840	C_20_H_32_	1942	1941	0.03
Hydrocarbon Monoterpene		61.82%				
Oxygenated Monoterpene		15.17%				
Hydrocarbon Sesquiterpene		8.18%				
Oxygenated Sesquiterpene		6.02%				
Diterpenes		0.03%				
Total identified		91.22%				
Yield (mg/100 g)		0.63%				

**Table 2:** The docking scores achieved by the major identified compounds against different enzymes.

Compound	α-Amylase	α-Glycosidase	Acetyl Cholinesterase
*α*-pinene	−4.62956142	−5.69745064	−4.83523035
*β*-pinene	−4.59063005	−5.4204216	−4.89209366
*α*-terpineol	−4.8473525	−6.05044317	−5.19867182
D-limonene	−4.59063005	−5.58485031	−5.07891607
myrcene	−4.67749071	−5.52444649	−5.16836262
*β*-(E)-ocimene	−4.53650188	−5.60387325	−5.58642292
(*E*)-*β*-caryophyllene	−5.61668322	−7.75139856	−6.75857782
Validation ligand	−5.83725119	−10.7614613	−8.74956799

## Data Availability

Data are available upon request from the first author.

## Sample Availability

Samples of the plant material and tested essential oil may be available from the authors.
